# Comparison of peripheral blood T, B, and NK lymphocytes between frontline medical workers for treating patients of COVID-19 and normal outpatient and emergency medical workers in China

**DOI:** 10.3389/fpsyt.2023.1165614

**Published:** 2023-04-19

**Authors:** Weijian He, Piyong Ma, Xiuying Li, Yali Wang, Yucheng Zhang

**Affiliations:** ^1^China-Japan Union Hospital of Jilin University, Changchun, China; ^2^The Third Norman Bethune Clinical College of Jilin University, Changchun, China; ^3^Department of Critical Care Medicine, China–Japan Union Hospital of Jilin University, Changchun, China; ^4^Scientific Research Center, China–Japan Union Hospital of Jilin University, Changchun, China; ^5^Department of Blood Transfusion, China–Japan Union Hospital of Jilin University, Changchun, China

**Keywords:** COVID-19, psychology stress, frontline medical workers, immunity, mental health

## Abstract

The outbreak of the novel coronavirus disease 2019 (COVID-19) has led to significant mental stress for frontline medical workers treating patients with confirmed COVID-19 in China. Psychological stress has an impact on the immune system. The number and percentage of lymphocyte subsets are standard indicators of cellular immune detection. Here, we reported the differences in CD3, CD4, CD8, CD19, and CD56 lymphocytes between 158 frontline medical workers and 24 controls from medical staffs of the outpatient and emergency departments. We found that frontline medical workers had significantly lower absolute values and percentages of CD19^+^ B cells, especially in the female and the aged ≥40 years subgroup. Stratification analysis showed that the absolute values of CD4^+^ T cells were significantly lower in the aged <40 years subgroup, while percentages of CD8^+^ T cells were lower and percentages of CD56^+^ NK cells were higher in the aged ≥40 years subgroup. In summary, this study suggests paying more attention to frontline medical workers’ mental health and immune function, and properly providing them with psychological interventions and measures of care.

## 1. Introduction

Novel coronavirus disease 2019 (COVID-19), caused by severe acute respiratory syndrome coronavirus 2 (SARS-CoV-2), is prevalent worldwide (1). COVID-19 is highly contagious and seriously harmful. Globally, there were more than 516 million COVID-19 cases that had been confirmed as of July 10, 2022, and there had been about 6.25 million fatalities overall, according to a report from the World Health Organization (WHO).

The immune system is the main mechanism by which the body defends itself against harmful pathogens. Lymphoid stem cells can differentiate into three main types of mature lymphocytes: B lymphocyte, T lymphocyte, and natural killer (NK) cell lineages (2). Measured as non-specific blood-validated markers, white blood cells (WBCs), and immune cell subsets play a role in the indication of immune function (3). The immune system is regulated by the neuroendocrine system. Previous studies have proved that acute stress, chronic stress, and job burnout have varying degrees of adverse effects on humoral immune function, NK cell, T lymphocyte function, and other immune parameters (4,5). A research on the occupational health status of emergency physicians in Japan showed that among overworked doctors, there were noticeable disparities in lymphocyte counts, CD4^+^ T cell counts, and NK cell activity; the low NK cell activity partially reflected the severity of the exhaustion brought on by the doctors’ overwork (6). Several studies of nurses performing shift work have shown that fatigue leads to lower NK cell activity and the deleterious effects on NK cell function depended on the degree of fatigue (7, 8). In a hospital in Guangxi, Cui et al. studied the immunological function of female oncology nurses and discovered that C3, C4, CD4^+^, and CD8^+^ T cells were substantially correlated with symptoms of burnout (9). In addition, a study of non-healthcare workers also found that the percentage of CD56^+^ cells decreased significantly when either the working hours per week increased or the sleep time decreased (10).

It is the substantial mental pressure brought to the frontline medical workers in China by the outbreak of the COVID-19 that attracts people’s attention. Survey results show a significant prevalence of symptoms of depression, anxiety, and distress among frontline healthcare professionals (11). A prospective study found that the workload of frontline medical workers is much larger than before participating in the fight against the COVID-19 pandemic. At the same time, various negative factors such as heavy assignments, social and professional isolation, a lack of time for physical activity and meditation, and compassion fatigue were reported among doctors, bringing them mental and psychological stress (12). Compared to non-frontline medical workers, frontline healthcare workers appear to experience much more anxiety, stress, and sleeplessness (11, 13). Furthermore, an immunological study has revealed that the lymphocyte count and lymphocyte ratio in the peripheral blood of frontline medical workers increased significantly after struggling with COVID-19, but gradually returned to normal several months later (14).

In the face of the above-mentioned unfavorable factors, we predict that the immune system of frontline medical workers will be affected, which is reflected in the fact that the lymphocyte subsets count and proportion are different from those of outpatient and emergency physicians in hospitals. However, the difference remains unclear. Here, we reported the differences of CD3, CD4, CD8, CD19, and CD56 lymphocytes between 158 frontline medical workers who returned to Changchun after supporting Wuhan and 24 outpatient and emergency physicians and nurses in the China-Japan Union Hospital of Jilin University during the same period. This study can provide a theoretical basis and ultimately help to provide appropriate psychological intervention for frontline medical workers.

## 2. Materials and methods

### 2.1. Participants

The present research involved 158 frontline medical workers from China-Japan Union Hospital who had given healthcare assistance to Wuhan COVID-19 patients as experimental group for 3 months, and 24 medical staffs of the outpatient and emergency department during the same period as control group. The exclusion criteria were as follows: (I) autoimmune disorders; (II) individuals suffering from tuberculosis, hepatitis B virus (HBV), AIDS (HIV), or hepatitis C virus (HCV); (III) patients had been treated with drugs that affect the immune system within 3 months; (IV) patients infected or had been infected with COVID-19. In addition, all subjects had not mental and psychological conditions such as depression and anxiety.

### 2.2. Apparatus and reagents

The antibody detection kit used for immunophenotype contains antibody Panel A (CD45-FITC/CD4-RD1/CD8-ECD/CD3-PC5 antibodies, LOT, 7536331) and antibody Panel B (CD45-FITC/CD56-RD1/CD19-ECD/CD3-PC5 antibodies, LOT, 7580257). Using a five-color FC 500 flow cytometry (Beckman Coulter) for sample collection.

### 2.3. Data collection and sampling processing

The demographic data were collected from the electronic health examination, including age, sex, occupation, and medical history. About 2 mL of blood was extracted from each fasting participant in EDTA-K2 tubes. Take 50 μL from anticoagulant whole blood samples and put them into two centrifuge tubes, marked as tubes A and B. Put 10 μL of antibody A and 10 μL of antibody B into two tubes, respectively. After stirring and mixing, incubate the cells in the darkness at ambient temperature for 15 min. Next, add 100 μL FCM Lysing solution to each tube. Incubate again for 10 min, then add 1 mL PBS and centrifuge at 1,500 r/min for 5 min. Lastly, discard the supernatant and add 500 μL PBS before detection by the flow cytometry within 24 h. Data were analyzed with Shortcut to CXP or Kaluza analysis software. Absolute values (cells/μL) = The absolute values of lymphocyte (cells/μL) × percentages of the lymphocyte subsets of interest × 1,000.

### 2.4. Statistical analyses

All statistical analyses were performed using data analysis software SPSS 26.0. Continuous variables were presented as mean ± SD, and units were expressed in the number of cells per microliter (cells/μL). The differences between the experimental and control groups, as well as those between frontline workers with different sexes or ages, were examined using the Student’s *t*-test. The significance threshold was *p* = 0.05.

## 3. Results

### 3.1. Demographic characteristics

Hundred and fifty eight front-line frontline healthcare professionals in total, along with 24 controls, were enrolled. Among 158 frontline workers, 37 were male and 121 were female. The average age of 158 frontline workers was 34.3 ± 5.8 years old, ranging in age from 23 to 53. Among 24 medical workers from normal outpatient and emergency departments, four were male and 20 were female. The age of 24 controls range from 28 to 53 years old and the mean age was 42.4 ± 8.8 years old. The demographic characteristics of all subjects are shown in Table 1.

**Table 1:** Characteristics of participants.

Group	*n*	Gender		Age (year)
		Male	Female	
Controls	24	4	20	42.4 ± 8.8
Experiments	158	37	121	34.3 ± 5.8

### 3.2. Comparison of absolute values and percentages of lymphocyte subsets between experimental and control groups

**Figure 1:**
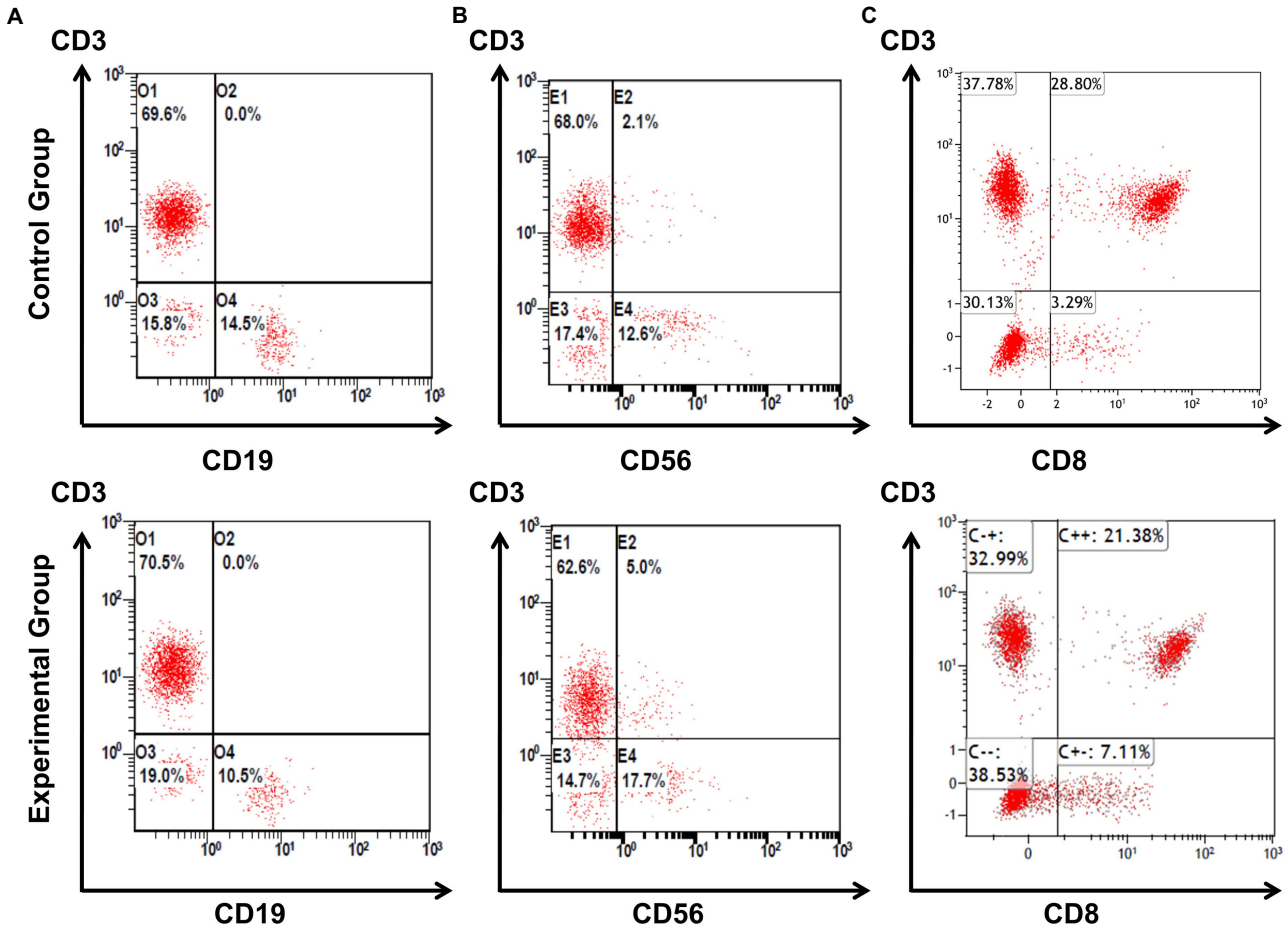
The main differences of percentages of lymphocyte subsets between experimental and control group. Frontline medical workers for treating patients of COVID-19 had significantly lower percentages of CD19^+^ B cells compared to control group, the representative results of flow cytometry are shown as line **(A)**. Compared with control group, stratification analysis showed that the percentages of CD56^+^ NK cells were higher in the aged ≥40 years subgroup, the representative results of flow cytometry are shown as line **(B)**. Stratification analysis also showed that the percentages CD8^+^ T cells were lower in the aged ≥40 years subgroup compared to control group, the representative results of flow cytometry are shown as line **(C)**.

Means, standard deviations, and percentages of lymphocyte subsets are shown in Table 2. The relative frequencies of each subpopulation in relation to the overall lymphocyte population are expressed as percentage. Compared with control group, absolute values and percentages of CD19^+^ B cells were significantly lower in experimental group (shown in Figure 1A). Stratification analysis based on gender and age showed that absolute values and percentages of CD19^+^ B cells were significantly lower only in the female subgroup and the aged ≥40 years subgroup. Absolute values and percentages of CD56^+^ NK cells were higher in experimental group, but the difference was not statistically significant. Compared with control group, stratification analysis based on age showed that percentages of CD56^+^ NK cells were significantly higher in the aged ≥40 years subgroup (shown in Figure 1B). Absolute values and percentages of CD4^+^T cells and CD8^+^T cells were lower in the experimental group, but the difference was not statistically significant. Stratification analysis based on age showed that absolute values of CD4^+^ T cells were significantly lower in the aged <40 years subgroup, while percentages of CD8^+^ T cells were significantly lower in the aged ≥40 years subgroup (shown in Figure 1C).

**Table 2:** The comparison between experimental group and control group (Mean ± SD, cells/μL).

Parameters	Age/gender	Control group	Experimental group
Lymphocytes		2,175 ± 475	2,047 ± 522
	Male	2,243 ± 623	2,275 ± 437
	Female	2,162 ± 460	1,977 ± 527
	<40 years	2,348 ± 483	2,042 ± 491
	≥40 years	2,052 ± 446	2,077 ± 707
CD3^+^		1,525 ± 368	1,433 ± 399
	Male	1,679 ± 521	1,553 ± 371
	Female	1,494 ± 339	1,396 ± 402
	<40 years	1,676 ± 409	1,451 ± 381
	≥40 years	1,417 ± 305	1,316 ± 497
CD4^+^		827 ± 224	740 ± 261
	Male	939 ± 296	782 ± 289
	Female	805 ± 209	727 ± 252
	<40 years	905 ± 248	744 ± 244 ^※^
	≥40 years	771 ± 196	714 ± 363
CD8^+^		585 ± 153	549 ± 195
	Male	579 ± 199	608 ± 189
	Female	586 ± 148	532 ± 194
	<40 years	610 ± 131	563 ± 190
	≥40 years	567 ± 169	463 ± 209
CD19^+^		284 ± 104	216 ± 82 ^※^
	Male	198 ± 18	246 ± 84
	Female	301 ± 106	207 ± 79 ^※^
	<40 years	261 ± 88	215 ± 81
	≥40 years	300 ± 114	227 ± 88 ^※^
CD56^+^		275 ± 107	288 ± 166
	Male	282 ± 105	343 ± 152
	Female	274 ± 110	271 ± 168
	<40 years	316 ± 95	274 ± 136
	≥40 years	246 ± 108	381 ± 284
CD4^+^/CD8^+^		1.46 ± 0.40	1.47 ± 0.65
	Male	1.64 ± 0.12	1.43 ± 0.68
	Female	1.42 ± 0.43	1.49 ± 0.65
	<40 years	1.49 ± 0.30	1.44 ± 0.62
	≥40 years	1.43 ± 0.47	1.73 ± 0.8
CD3^+^%		70.1 ± 5.7	70.0 ± 7.8
	Male	74.5 ± 3.6	68.1 ± 8.2
	Female	69.2 ± 5.7	70.6 ± 7.6
	<40 years	71.3 ± 7.5	71.0 ± 7.0
	≥40 years	69.2 ± 4.2	63.8 ± 10.1
CD4^+^%		37.8 ± 5.0	36.0 ± 7.6
	Male	41.6 ± 1.9	34.1 ± 9.2
	Female	37.1 ± 5.1	36.6 ± 6.9	
	<40 years	38.2 ± 4.3	36.3 ± 7.0
	≥40 years	37.6 ± 5.5	34.0 ± 10.5
CD8^+^%		27.2 ± 5.3	27.0 ± 7.0
	Male	25.6 ± 2.8	26.7 ± 6.6
	Female	27.5 ± 5.7	27.1 ± 7.1
	<40 years	26.4 ± 5.6	27.6 ± 6.7
	≥40 years	27.7 ± 5.2	22.9 ± 7.3 ^※^
CD19^+^%		13.2 ± 4.4	10.7 ± 3.4 ^※^
	Male	9.3 ± 2.5	10.9 ± 3.2
	Female	13.9 ± 4.4	10.7 ± 3.5 ^※^
	<40 years	11.2 ± 3.9	10.6 ± 3.3
	≥40 years	14.6 ± 4.4	11.4 ± 4.3 ^※^
CD3-CD56^+^%		12.6 ± 4.2	13.9 ± 6.4
	Male	12.6 ± 4.3	15.2 ± 6.5
	Female	12.6 ± 4.3	13.5 ± 6.3
	<40 years	13.4 ± 3.5	13.3 ± 5.7
	≥40 years	12.0 ± 4.7	17.6 ± 9.1 ^※^

※ *p* < 0.05.

### 3.3. Gender distribution of lymphocyte subsets in experimental group

Gender distribution of lymphocyte subsets in experiments are shown in Table 3. Absolute values of lymphocytes and CD3^+^ T cells were significantly lower in the female subgroup (1,977 ± 527 and 1,396 ± 402/μL), compared with the male subgroup (2,275 ± 437 and 1,553 ± 371/μL). Likewise, absolute values of CD8^+^ T cells, CD19^+^ B cells and CD56^+^ NK cells were significantly lower in the female subgroup (532 ± 194, 207 ± 79, and 271 ± 168/μL), compared with the male subgroup (608 ± 189, 246 ± 84, and 343 ± 152/μL). Besides, the value of CD4^+^/CD8^+^ was higher in the female subgroup (1.49 ± 0.65) when compared with the male subgroup (1.43 ± 0.68), but the difference was not statistically significant.

**Table 3:** Absolute values and percentages of lymphocyte subpopulations in experimental group by sex (Mean ± SD, cells/μL).

Parameter	Male (*n* = 37)	Female (*n* = 121)
Lymphocytes	2,275 ± 437	1,977 ± 527^※^
CD3^+^	1,553 ± 371	1,396 ± 402^※^
CD3^+^%	68.1 ± 8.2	70.6 ± 7.6
CD3^+^/CD4^+^	782 ± 285	727 ± 252
CD3^+^/CD4^+^%	34.1 ± 9.2	36.6 ± 6.9
CD3^+^/CD8^+^	608 ± 189	532 ± 194^※^
CD3^+^/CD8^+^%	26.7 ± 6.6	27.1 ± 7.1
CD19^+^	246 ± 84	207 ± 79^※^
CD19^+^%	10.9 ± 3.2	10.7 ± 3.5
CD3^−^/CD56^+^	343 ± 152	271 ± 168^※^
CD3^−^/CD56^+^%	15.2 ± 6.5	13.5 ± 6.3
CD4^+^/CD8^+^	1.43 ± 0.68	1.49 ± 0.65

※ *p* < 0.05.

### 3.4. Age distribution of lymphocyte subsets in experimental group

Age distribution of lymphocyte subsets in experiments are shown in Table 4. Absolute values of CD8^+^ T cells, percentages of CD3^+^ T cells and CD8^+^T cells were significantly lower in the aged ≥40 years subgroup (463 ± 209/μL, 63.8 ± 10.1%, and 22.9 ± 7.3%), compared with the aged <40 years subgroup (563 ± 190/μL, 71.0 ± 7.0%, and 27.6 ± 6.7%). However, absolute values and percentages of CD56^+^ NK cells were significantly higher in the aged ≥40 years subgroup (381 ± 284/μL and 17.6 ± 9.1%), compared with the aged <40 years subgroup (274 ± 136/μL and 13.3 ± 5.7%).

**Table 4:** Absolute values and percentages of lymphocyte subpopulations in Experiment group by age group (Mean ± SD, cells/μL).

Parameter	Age < 40 years (*n* = 137)	Age ≥ 40 years (*n* = 21)
Lymphocytes	2,042 ± 491	2,077 ± 707
CD3^+^	1,451 ± 381	1,316 ± 497
CD3^+^%	71.0 ± 7.0	63.8 ± 10.1^※^
CD3^+^/CD4^+^	744 ± 244	714 ± 363
CD3^+^/CD4^+^%	36.3 ± 7.0	34.0 ± 10.5
CD3^+^/CD8^+^	563 ± 190	463 ± 209^※^
CD3^+^/CD8^+^%	27.6 ± 6.7	22.9 ± 7.3^※^
CD19^+^	215 ± 81	227 ± 88
CD19^+^%	10.6 ± 3.3	11.4 ± 4.3
CD3^−^/CD56^+^	274 ± 136	381 ± 284^※^
CD3^−^/CD56^+^%	13.3 ± 5.7	17.6 ± 9.1^※^
CD4^+^/CD8^+^	1.44 ± 0.62	1.73 ± 0.80

※ *p* < 0.05.

## 4. Discussion

This study reported the changes in absolute values and percentages of T lymphocytes and their subpopulations, as well as B lymphocytes and NK cells of frontline medical workers for treating patients of COVID-19 compared to normal outpatient and emergency physicians. We found that frontline medical workers had significantly lower absolute values and percentages of CD19^+^ B cells, especially in females and aged ≥40 years subgroup. Stratification analysis showed that the absolute values of CD4^+^ T cells were significantly lower in the aged <40 years subgroup, while percentages of CD8^+^ T cells were lower and percentages of CD56^+^ NK cells were higher in the aged ≥40 years subgroup. Besides, we found that the changes were more obvious in females and the aged ≥40 years among frontline medical workers. Furthermore, no medical workers had been identified to be infected with COVID-19 or to be suffering from mental disorder.

The outbreak of COVID-19 has brought a series of psychological and spiritual stress to frontline medical workers (11, 15). Zhang et al. found that unlike non-healthcare staffs, healthcare staffs gained a higher prevalence of insomnia, anxiety, and depression symptoms (16). And Lai et al. reported that depression represented the highest at 50.4% of the total number of healthcare workers, while anxiety symptoms and insomnia accounted for 44.6% and 34.0% of the total number of healthcare workers, respectively (1). A study in Ecuador also reported that 66% of the subjects that manifested psychological distress, especially women with COVID-19 symptoms and previous exposure to infected patients or objects (17). These studies show us that frontline medical workers have huge psychological stress. The sympathetic nervous system and the hypothalamic–pituitary–adrenal axis, which are influenced by mental distress, that upregulate the levels of catecholaminergic neurotransmitters and corticosterone, leading to active immune responses and leukocytes redistribution, revealed by prior research (18–21). We suggest that the changes in the absolute values and proportions of immune cells may be caused by a series of psychological stress, and such changes may increase the risk of contracting the coronavirus or other diseases, such as inflammatory bowel disease (22). In addition, being in a state of psychological stress for a long time is not conducive to dealing with work affairs and serving patients. Therefore, more attention should be paid to the mental health and immunity level of frontline workers, and appropriate psychological interventions should be provided.

B lymphocytes participate in the process of clearing pathogens by secreting a variety of antibodies, such as IgM, IgG, etc. (23). In addition, B lymphocytes can also function without antibodies and play an important role in immune system development and maintenance (24). When the body is under appropriate stress, the brain can affect the formation of plasma cells and regulate humoral immunity by activating the spleen via the brain-spleen axis (25–28). However, chronic stress caused reduction in circulating B cells, T cells, and large granular lymphocytes and decreased natural killer cell activity (28–30). In this study, the absolute values and proportions of CD19^+^ B cells in the frontline medical workers were significantly reduced. This may be due to the long-term busy and stressful working environment, which inhibited humoral immunity.

Many studies showed that there are sex differences in stress responses (31). In the face of stressors, testosterone is negatively correlated with cortisol levels in men, while estrogen in women stimulates the output of the HPA axis (21). Women responded to acute stressors in a proinflammatory fashion but experienced greater suppression of the immune system under chronic stress than men (32). In the current study, the changes in immune cells of frontline medical workers were more obvious in women, and the values and proportions of some lymphocyte subsets were lower than those of males in the same period, which may be more susceptible to disease by contrast. Previous studies showed that chronic stress reduces the values and proportion of nature killer cells (10, 33). The present study found that the proportion of CD56+ NK cells in the aged ≥40 years subgroup was significantly increased, and the absolute value was increased but there was no significant difference. The inconsistency of these results may be attributed to differences in the inclusion criteria, sample size, age distribution, etc. of the participants.

The limitations of our study mainly include screening criteria, sample size, and detection content. First of all, we did not conduct self-administered questionnaires and other methods on frontline medical workers before the research to assess their psychological outcomes. Secondly, the sample of normal outpatient and emergency medical workers during the same period selected was relatively small. Thirdly, our study did not count neutrophils and monocytes, which are the immune cells affected by chronic stress and can reflect the level of stress. Moreover, it would be more complete and helpful for this report if the function of T cells and NK cells, and common cytokine levels in the peripheral blood could be detected.

## 5. Conclusion

The results of the present study indicated that the changes of T lymphocytes and their subpopulations, as well as B lymphocytes and NK cells, were found in frontline medical workers providing support for Wuhan COVID-19 patients, especially in females and physicians over 40 years old. Those may be attributed to psychological stress such as work-related stress. As a result, we suggest paying more attention to the psychological health and immune function of frontline medical staffs, and properly providing them with psychological interventions and measure of care. Here are some of our suggestions: (I) Government departments should adjust and improve the current work arrangements to avoid overloading medical personnel. (II) It is necessary for the relevant authorities to give material support to protect medical workers’ interests as soon as possible, such as the supply of protective materials and daily necessities. (III) Hospitals should routinely conduct physical examinations and make mental health evaluations for medical staffs.

## Data availability statement

The original contributions presented in the study are included in the article/supplementary material, further inquiries can be directed to the corresponding authors.

## Ethics statement

The studies involving human participants were reviewed and approved by the Research Ethics Committee of China-Japan Union Hospital of Jilin University. The patients/participants provided their written informed consent to participate in this study.

## Author contributions

WH and PM collected the literature and wrote the manuscript. XL edited the manuscript. YW and YZ wrote, conceived, and reviewed the manuscript critically. All authors contributed to the article and approved the submitted version.

## Funding

This research was funded by Health and Scientific Research Top-Notch Talent Project of Jilin Province (2022SCZ07), Health and Scientific Research Talent Project of Jilin Province (2020SCZ50), Foundation of Jilin Provincial Science and Technology Department (20200404115YY), Science and Technology Plan Projects of Jilin Province (20200201539JC), Jilin University Project (2019YX396), and National Natural Science Foundation of China (82000765).

## Conflict of interest

The authors declare that the research was conducted in the absence of any commercial or financial relationships that could be construed as a potential conflict of interest.

## Publisher’s note

All claims expressed in this article are solely those of the authors and do not necessarily represent those of their affiliated organizations, or those of the publisher, the editors and the reviewers. Any product that may be evaluated in this article, or claim that may be made by its manufacturer, is not guaranteed or endorsed by the publisher.
